# Five low energy phosphorene allotropes constructed through gene segments recombination

**DOI:** 10.1038/srep46431

**Published:** 2017-04-27

**Authors:** Chaoyu He, ChunXiao Zhang, Chao Tang, Tao Ouyang, Jin Li, Jianxin Zhong

**Affiliations:** 1 Hunan Key Laboratory for Micro-Nano Energy Materials and Devices, Xiangtan University, Hunan 411105, P. R. China; 2 School of Physics and Optoelectronics, Xiangtan University, Xiangtan 411105, China

## Abstract

Based on the crystal structures of the previously proposed low energy *η***-P** and *θ***-P**, five new phosphorene allotropes were predicted through gene segments recombination method. These five new phosphorene allotropes are confirmed dynamically stable and energetically more favorable than their parents (*η***-P** and *θ***-P**). Especially, the **XX-XX** type **G1-P** is confirmed energetically more favorable than most of all the previously proposed phosphorene allotropes, including black phosphorene and blue phosphorene, which is highly expected to be synthesized in future experiment through vapor deposition or epitaxial growth method like blue *β***-P**. The calculated results also show that such a new promising phosphorene allotrope **G1-P** is a potential candidate for application in nano-electronics according to its middle band gap of about 1.491 eV from DFT-HSE06 calculation.

Black phosphorene (*α***-P**)^1^,^2^, a new atomic thin two-dimensional material, was successfully exfoliated from its three-dimensional counterpart black phosphorus through mechanical method in 2014. As a new-star in two dimensional materials family, black phosphorene is considered as a formidable competitor to graphene and other two-dimensional materials for application in nano-electronic fields due to its significant band gap^3^,^4^ and high carrier mobility^2^. Thus, synthesis of such a new material with low-cost and high-yielding deposition methods, such as chemical or physical vapor deposition, is highly expected.

Usually, the possible crystal structure for a quasi two dimensional phosphorene synthesized from deposition methods always depends on the substrate, pressure, temperature and other experimental conditions. Theoretically, many potential quasi two dimensional phosphorene allotropes have been proposed in the past few years, including the black *α***-P**^1^,^2^, blue *β***-P**^5^,^6^, γ**-P**^7^,*δ***-P**^7^, *θ*_0_**-P**^8^ and red phosphorene^9^ in a full 6–6 ring atomic thin layer, the diatomic thin layers *η***-P** and *θ***-P** with pentagons^10^, as well as some other atomic thin layers with 4–8, 5–7, 5–8 or 3–12 type topological characteristics^7^,^10^,^11^. In these phosphorene allotropes, the most stable five ones are stirrup black *α***-P**, tricycle red phosphorene, diatomic thin *θ***-P**, chair blue *β***-P** and diatomic thin *η***-P**, in which that the third stable chair blue phosphorene have been synthesized recently^12^.

Very recently, twenty one quasi two dimensional porous phosphorene allotropes were proposed^13^,^14^ through topological modeling method and investigated by first principles calculations. These new potential phosphorene allotropes showing turnable energy band gaps, which provide us many potential structural candidates to understand future experiment. In this letter, we apply the gene segments recombination method^15^,^16^ to the previously proposed diatomic thin layers *θ***-P** (provide us **XX** gene segment) and *η***-P** (provide us **XY** gene segment) and find five new structural stable phosphorene boys (**XX-XY** or **XY-XY**) and girls (**XX-XX**) with distinct and fascinating two dimensional topology patterns (See in Fig. 1). Density functional theory (DFT) based first-principles method is employed to investigate the structures, energetic stabilities, dynamical stabilities and electronic properties of these five new possible phosphorene allotropes. Our results show that these five new phosphorene allotropes are dynamically stable and three of them are more favorable than their parents in energy. Especially, the **XX-XX** type girl **G1-P** is confirmed energetically more favorable than all the previously proposed phosphorene allotropes, including its mother *θ***-P** and the experimentally viable black *α***-P** and blue *β***-P**. Such a result suggests that **G1-P** is a promising new two dimensional material with high probability to be synthesized in future vapor deposition experiments. The results also show that **G1-P** is a potential candidate for application in nano-electronics according to its middle band gap of 1.491 eV from DFT-HSE06 calculation.

## Results and Discussion

### Crystal structures

**Figure 1:**
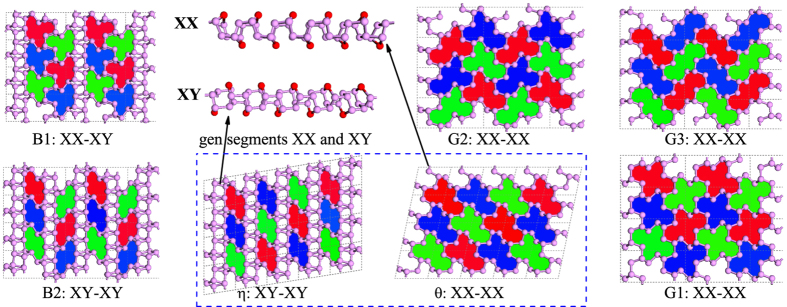
Top views and corresponding topological patterns of diatomic thin *η***-P**, *θ***-P** and their descendants **G1**, **G2**, **G3**, **B1** and **B2**. The two gene segments **XX** and **XY** abstracted form *θ***-P** and *η***-P**, respectively, are also shown here.

As shown in Fig. 1 and Fig. S1, two structural segments can be abstracted from the previously proposed low energy diatomic thin phosphorene allotropes *θ***-P** and *η***-P**. They are named as **XY** and **XX**, respectively, according to their structural characteristics. We can see that the **XY** (**XX**) gene is consisted of **X-Y** (**X-X**) stacked bilayer (we classify *θ***-P**, *η***-P** and their posterities to category of bilayers) of armchair phosphorus chains connecting by inter-chain phosphorus atoms (highlighted as red balls). Based on gene segments **XX** and **XY**, we propose five new phosphorene allotropes with remarkable energetic stabilities. In fact, infinite allotropes can be constructed by **XX** and **XY** gene segments, but we considered only the situations containing two gene segments per unit cell here. We define those containing only **XX** gene in their bodies as female girls and name them as **G1**, **G2** and **G3**. Those containing **XY** gene are correspondingly defined as male boys and they are named as **B1** and **B2** in our work. In Fig. 1, the top views of **G1**, **G2**, **G3**, **B1**, **B2** and their parents *θ***-P** and *η***-P** are shown.

From Fig. 1, we can also see that the stacking type between two adjacent **XX** and/or **XY** gene segments in each phosphorene allotrope can form individual tiling pattern, which provides us helpful topology characteristics to distinguish them. Only seven staking types are considered in present work, they are *θ***-P**, **G1**, **G2** and **G3** of **XX-XX**, *η***-P** and **B2** of **XX-XY** and **B1** of **XY-XY**. Other stacking types have also been considered in testing task but they possess relatively high energy, and thus are not considered in our present work. These five new allotropes contain two gene segments in their crystalline lattice, which are different to their parents those contain only one gene segment in their crystalline cell. Detail structural information, such as crystalline view from different directions, symmetry group, lattice constants and atomic positions, of these phosphorene allotropes are prepared in the Supplementary file. One can reproduces these phosphorene crystals according to their crystalline information to further study their structures or investigate their other physical properties.

### Energetic and dynamic stabilities

**Figure 2:**
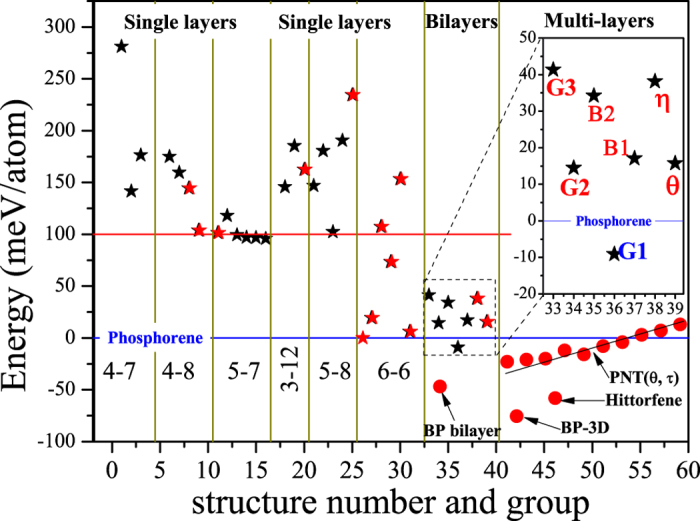
Total energies per atom of possible phosphorene allotropes (red five-point stars mean phosphorene allotropes predicted previously, black ones mean those discovered in our present work, red-solid circles represent Hittorfene^17^, black phosphorene bilayers, black phosphorus (3D) and phosphorus nanotube arrays PNT(*θ,τ*)^19^ considered in for comparison) are summarized here in different categories. The total energy of *α***-P** is set to be zero as reference. From these results, we can see that G1 is energetically more stable than most of all the previously proposed 2D phosphorene allotropes.

To evaluate the relatively energetic stabilities between these phosphorene allotropes, we calculated their total energies relative to that of the black *α***-P**. All previously proposed phosphorene allotropes and some other possible structures (mutations from the previously proposed ones) were also considered in for comparison. The results are classified according to their structural characteristics and plotted in Fig. 2 Our calculations show that the total energy of *θ***-P** and *β***-P** relative to *α***-P** are 15 meV/atom and 19 meV/atom, respectively, which are good consistent with those reported in previous work^10^ and are reliable according to our testing results for cut off energy and K-mesh (See Fig. S2). From Fig. 2, we can see that some previously proposed phosphorene allotropes (marked as red five-pointed stars) are not the most stable one in their corresponding categories. For example, in single-layered 5–7, 3–12 and 5–8, we find some more favorable candidates. They are marked as black five-pointed stars in Fig. 2 and please see them for more detail in the Supplementary Figures S3–S7 and Tables S2–S7.

As displayed in Fig. 2 in the category of bilayers, the five new allotropes (**G1**, **G2**, **G3**, **B1** and **B2**) constructed through gene segment recombination in our present work show remarkable stability. The total energies of **G1**, **G2**, **G3**, **B1**, **B2**, *η***-P** and *θ***-P** relative to *α***-P** are −9 meV/atom, 14 meV/atom, 41 meV/atom, 17 meV/atom, 34 meV/atom, 38 meV/atom and 15 meV/atom, respectively. We can see that **G1** is more favorable than its parents and **G2**, **G3**, **B1** as well as **B2** are energetically comparable to their parents. Especially, the XX-XX type **G1** is more favorable than most of all (except the multilayered phosphorene bilayer and Hittorfene^17^) the previously proposed two dimensional phosphorene allotropes, including the experimentally achieved black *α***-P** and blue *β***-P**. Although the energy differences between some phosphorene structures are lie in DFT undistinguishable range (level in meV), we still believe that we have found a new phosphorene allotrope **G1** with excellent stability more favorable than black *α***-P**, according to our testing results (see in Supplementary Fig. S2) and the fact that the bilayer black *α***-P** and the 3D black phosphorus are more favorable than single layer black *α***-P** due to the impressive inter-layer vdW interaction. We understand such a remarkable energetic stability of **XX-XX** type **G1** is due to its proper staking manner between the two adjacent **XX** gene chains, in which the inter-chains vdW interactions cause a sizable energy reduction. Such a suppose can also be proofed in our resent work^18^ about assembling of the 1D phosphorus nanotubes^19^ into 2D planar structures (PNT(*θ,τ*)) in different stack manners, which will cause remarkable energy release and result in different stability according to the stacking manner. As listed in Table 1, our HSE06 results also confirm such an energy sequence in these phosphorene allotropes and the fact that **XX-XX** type **G1** is more favorable than black *α***-P**. We believe that there are still many other new forms of phosphorene allotropes will be predicted more favorable than black *α***-P** and **G1** in future, such as the one called as Hittorfene proposed by G. Schusteritsch very recently^17^ and those phosphorus nanotube arrays PNT(*θ, τ*) proposed by our group^18^. Further theoretical and experimental efforts are expected to be paid on searching for them and synthesizing them^19^,^20^.

**Figure 3:**
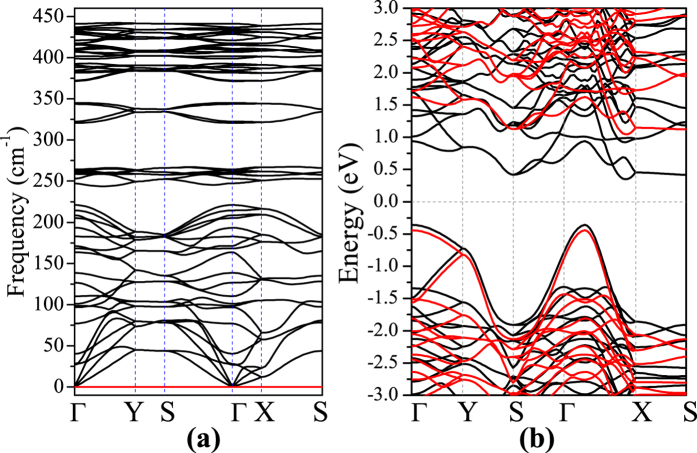
Phonon band structure (**a**) and electron band structure (**b**) of **G1** calculated form both DFT (black solid line) and HSE06 (blue solid line) methods.

From the view of thermodynamics, low energy generally means high probability to be synthesized in experiments if the system is dynamically possible. The **XX-XX** type **G1** with remarkable stability exceeding black *α***-P** is expected to be synthesized in future vapor deposition method. We then care about the dynamical stabilities of these five new phosphorene allotropes to confirm the possibility of to be synthesized. We evaluate their dynamical stability through simulate their vibrational property. As shown in Fig. 3(a), the phonon band structure of **G1** phosphorene is free of soft modes associated with structural instabilities. We have also checked the whole Brillouin Zone and find no any imaginary states in its phonon density of states (See Fig. S9). Such results show that allotrope **G1** is dynamically stable. The dynamical stabilities of the other four new phosphorene allotropes (**G2**, **G3**, **B1** and **B2**) are also confirmed positive according to their phonon band structures and phonon density of states as shown in Supplementary Figs S8 and S9. To confirm the thermal stability of these five new phosphorene allotropes, we have also performed Born-Oppenheimer molecular dynamics (BOMD) simulation. The corresponding results shown in Figs S11 and S12 indicate that these five new phosphorene allotropes can keep intact at 300 K. In view of the fact that *θ***-P** and *η***-P** can stable even at 700 K^10^, we believe that their transformers **G1**, **G2**, **G3**, **B1** and **B2** are still intact at more higher temperature.

### Electronic properties and Raman shift

We then care about the fundamental electronic property of such a promising new phosphorene allotrope **G1**. The calculated band structure of allotrope **G1** is investigated in both DFT and HSE06 methods. As shown in Fig. 3(b), we can see that allotrope **G1** is an indirect band gap semiconductor with band gap of 1.491 eV in HSE06 level, which is slightly lower than that of black *α***-P**. Our HSE06 calculated band gap for black *α***-P** is 1.533 eV, which is good consistent with those reported in previous reports^13^,^17^,^21^. Such a promising new phosphorene **G1** with middle band gap is a good candidate for application in nano-electronics. The band structures of other four allotropes (*η***-P**, *θ***-P**, **G2**, **G3**, **B1** and **B2**) are also investigated by both DFT and HSE06 methods and prepared as Supplementary in Fig. S9. From these results, we can see that all of these new phosphorene allotropes are indirect band gap semiconductors with middle band gaps, which are proper for semiconductor application.

**Figure 4:**
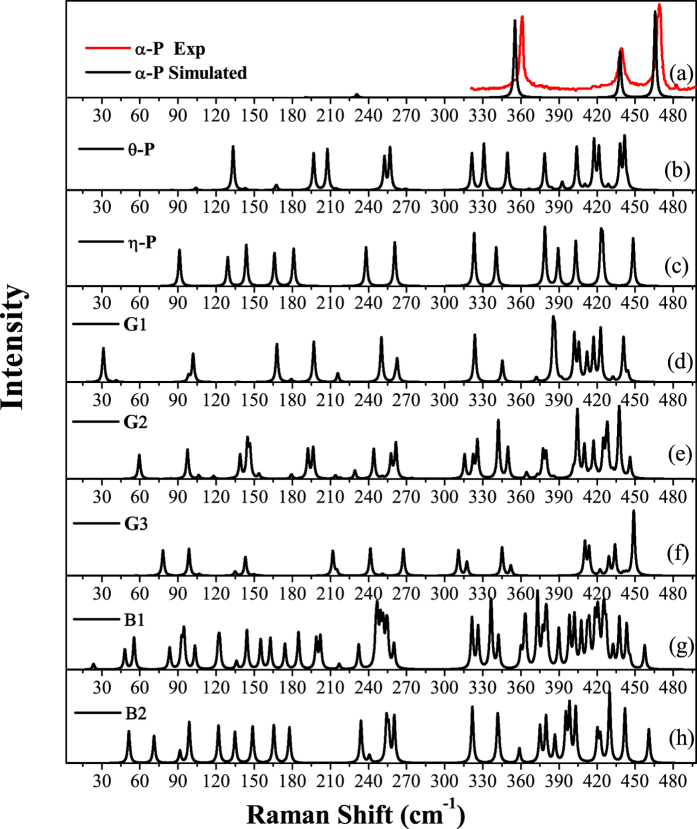
Raman Shift spectra of the previously proposed black *α***-P** (**a**), *θ***-P** (**b**) and *η***-P**, as well as the new **G1** (**d**), **G2** (**e**), **G3** (**f**), **B1** (g) and **B2** (**h**). The peak height is in the logarithmic scale and it is smeared by Lorentzian function with a 2 cm^−1^ width.

Finally, we simulated the Raman shift spectra of these five new phosphorene allotropes and compared them with those of their parents and black *α***-P**. These Raman shift results are helpful in experiment to differentiate them from each other. As shown in Fig. 4(a), the black solid line and the red solid line are the Raman shift results of *α***-P** from previous experiment^1^ and present simulation, respectively. We can see that the simulated result matches well with the experimental result^1^, which confirms that our method is reliable. The simulated Raman spectra of the previously proposed *θ***-P** and *η***-P** are plotted in Fig. 4(b) and (c), respectively, for comparison. We can see that they are very different form that of the black *α*-P, which indicates that we can easily distinct them out from black *α***-P** through measuring their Raman spectra in experiment if they are synthesized. The Raman shift results of **G1**, **G2**, **G3**, **B1** and **B2** are plotted in Fig. 4(d–h), respectively. They are very different from those of their parents (*θ***-P** and *η***-P**) and black *α***-P**, and also different from each other. These results provide useful data for experimentally differentiating these potential quasi two dimensional phosphorene allotropes in future.

## Conclusions

In summary, gene segments recombination method was applied to recently proposed diatomic thin layers *η***-P** and *θ***-P** and five new structural stable phosphorene boys (**XX-XY** or **XY-XY**) and girls (**XX-XX**) with distinct and fascinating two dimensional topology patterns were proposed to extend phosphorene allotropes family. Our first-principles calculation results show that these five new phosphorene allotropes are dynamically stable and show remarkable energetic stability. The **XX-XX** type girl **G1-P** is confirmed energetically more favorable than most of all the previously proposed phosphorene allotropes, including the experimentally achieved black *α***-P** and blue *β***-P**. Such a result suggests that **G1-P** is a promising new two dimensional material with high probability to be synthesized in future vapor deposition experiments, which is a potential candidate for application in nano-electronics according to its middle band gap of 1.491 eV from DFT-HSE06 calculation. The experimentally detectable Raman shift properties of these five new phosphorene allotropes are investigated and compared with those of their parents and black *α***-P**, which are very useful for future distinguish them from each other in future experiment.

## Methods

Our calculations of structural optimization and properties investigations are carried out by using the density functional theory (DFT) within generalized gradient approximations (GGA)^22^ as implemented in Vienna ab initio simulation package (VASP)^23^,^24^. The interactions between nucleus and the 3s^2^3p^3^ valence electrons of phosphorus atoms are described by the projector augmented wave (PAW) method^25^,^26^. To ensure the accuracy of our calculations, a plane-wave basis with a cutoff energy of 500 eV is used to expand the wave functions and the Brillouin Zone (BZ) sample meshes are set to be dense enough (less than 0.21 Å^−1^) for each system considered in present work (these settings are set according the convergence test for some important parameters based on the black *α***-P** and **G1-P**). The structures of these five new phosphorene allotropes and some other reference systems considered in present work are fully optimized up to the residual force on every atom less than 0.001 eV/Å. In such a structural optimization process, the optimized exchange van der Waals functional (optB88-vdW)^27^,^28^ is applied to take into account van der Waals interactions. Especially, to gain more reasonable energy band gap of these phosphorene allotropes, the hybrid functional method (HSE06)^29^ is considered in the processes of properties investigations after structural optimization. We also simulated the vibrational properties of the five new phosphorene allotropes proposed in our present work through the PHONON package^30^ with the forces calculated from VASP to confirm their dynamical stabilities. For the purpose of providing experimentally detectable property to differentiate these possible phosphorene allotropes, we simulated their Raman shift through CASTEP software^31^.

## Additional Information

**How to cite this article:** He, C. *et al*. Five low energy phosphorene allotropes constructed through gene segments recombination. *Sci. Rep.* **7**, 46431; doi: 10.1038/srep46431 (2017).

**Publisher's note:** Springer Nature remains neutral with regard to jurisdictional claims in published maps and institutional affiliations.

## Supplementary Material

Supplementary Information

## Figures and Tables

**Table 1:** Layer Thickness (LT: Å), total energy (E: meV/atom) relative to *α*-P and energy band gaps (Eg: eV) for different phosphorene allotropes simulated from both DFT and HSE method.

system	LT	E-DFT	E-HSE	Eg-DFT	Eg-HSE
*α***-P**	2.126	0	0	0.781	1.533
*α***-P** bilayer	7.711	−46	—	—	—
Hittorfene	9.344	−58	—	—	—
*η***-P**	3.484	38	56	0.913	1.721
*θ***-P**	3.541	15	33	1.183	1.995
**G1-P**	3.478	−9	−4	0.707	1.491
**G2-P**	3.464	14	33	1.124	1.931
**G3-P**	3.439	41	58	1.162	1.977
**B1-P**	3.497	17	34	0.761	1.526
**B2-P**	3.492	34	52	0.899	1.791
