# Supplementary material for: Five low energy phosphorene allotropes constructed through gene segments recombination
Source: Sci Rep. 2017 Apr 27;7:46431. doi: 10.1038/srep46431 (PMC5406830; doi:10.1038/srep46431)
Supplement: Supplementary Information [file srep46431-s1.pdf]

Supplementary for “Five low energy phosphorene allotropes constructed through gene segments recombination”

Chaoyu He*, ChunXiao Zhang, Chao Tang*, Tao Ouyang, Jin Li and Jianxin Zhong

Hunan Key Laboratory for Micro-Nano Energy Materials and Devices, Xiangtan University, Hunan 411105, P. R. China;

School of Physics and Optoelectronics, Xiangtan University, Xiangtan 411105, China.

Here we provide a supplementary file including 1) perspective crystalline views of **η-P, θ-P, G1, G2, G3, B1** and **B2** from different directions, 2) testing results based on allotrope **G1** and black **α-P** for cutoff energy and K-mesh, 3) crystal structures, relative energies, and band structures of phosphorene allotropes in categories 4-7, 3-12, 4-8, 5-8 and 5-7, 4) phonon band structures and density of states for allotropes **G1, G2, G3, B1** and **B2**, 5) electronic band structures of allotropes **η-P, θ-P**, **G2, G3, B1** and **B2**, 6) as well as lattice constants and atomic positions for **G1, G2, G3, B1** and **B2** in format of VASP-POSCAR.

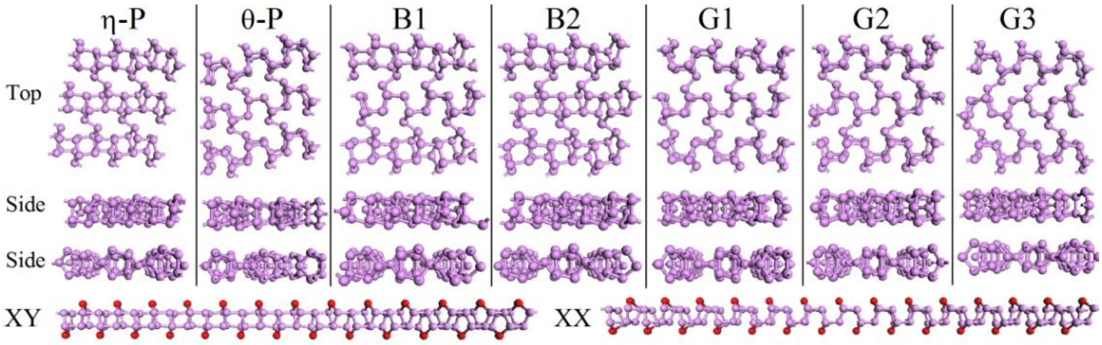

Fig. S1. Perspective top and side views of allotrope **η-P, θ-P, B1, B2, G1, G2** and **G3** and the corresponding **XY** and **XX** gene segments are shown.

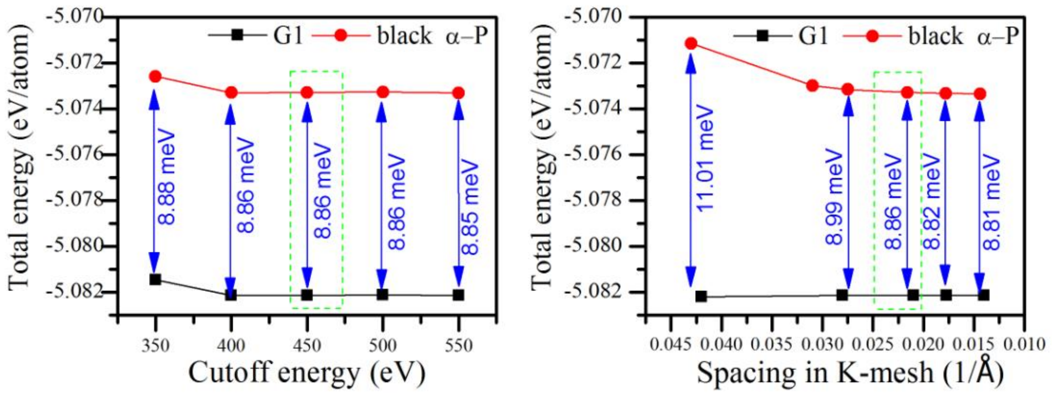

Fig. S2. Testing results based on allotrope **G1** and black **α-P**: dependence of total energy on cutoff energy (left) and on sample K-mesh (right).

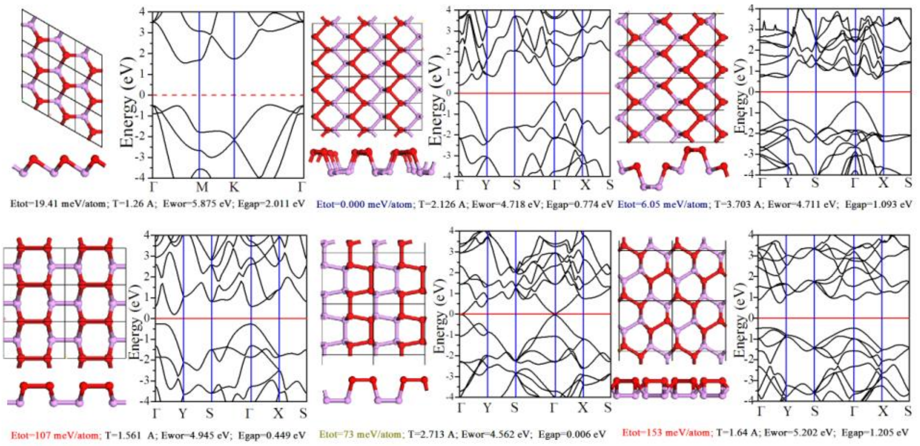

Fig. S3 The optimized crystal structures, relative energies (respect to **α-P**, meV/atom), band structures and band gaps of single-layered 6-6 phosphorene allotropes based on DFT-calculation.

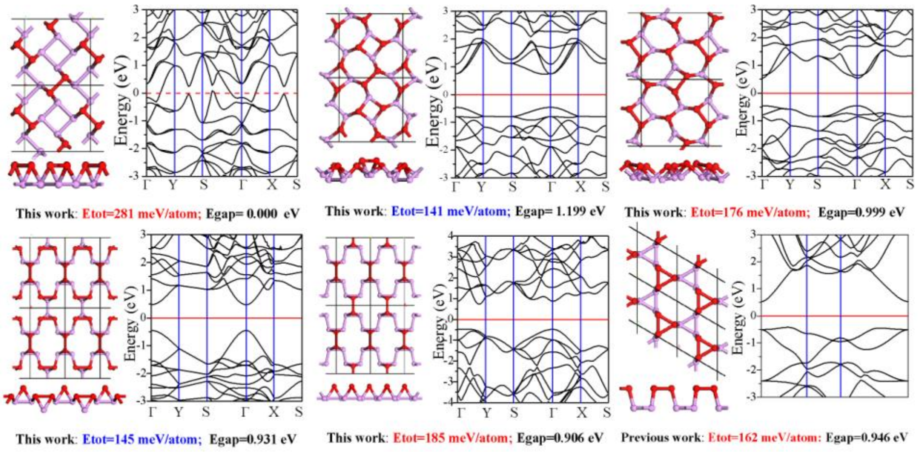

Fig. S4 The optimized crystal structures, relative energies (respect to **α-P**, meV/atom), band structures and band gaps of single-layered 4-7 (top 3) and 3-12 (bottom 3) phosphorene allotropes based on DFT-calculation.

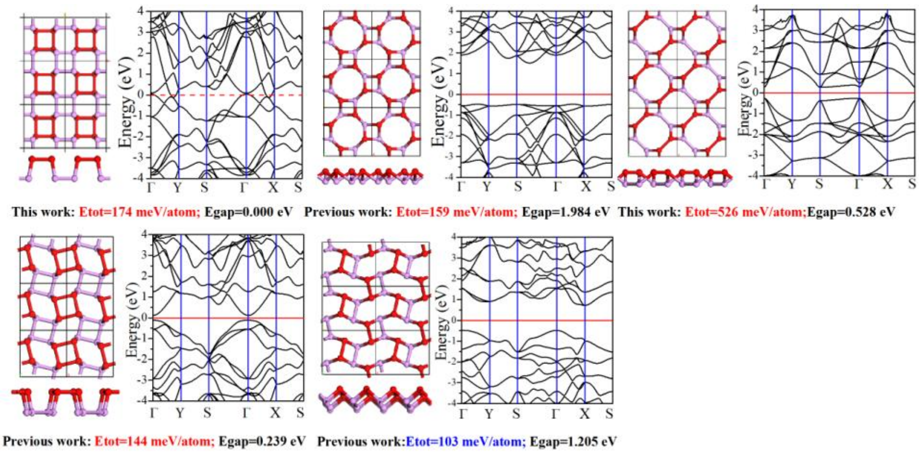

Fig. S5 The optimized crystal structures, relative energies (respect to **α-P**, meV/atom), band structures and band gaps of single-layered 4-8 phosphorene allotropes based on DFT-calculation.

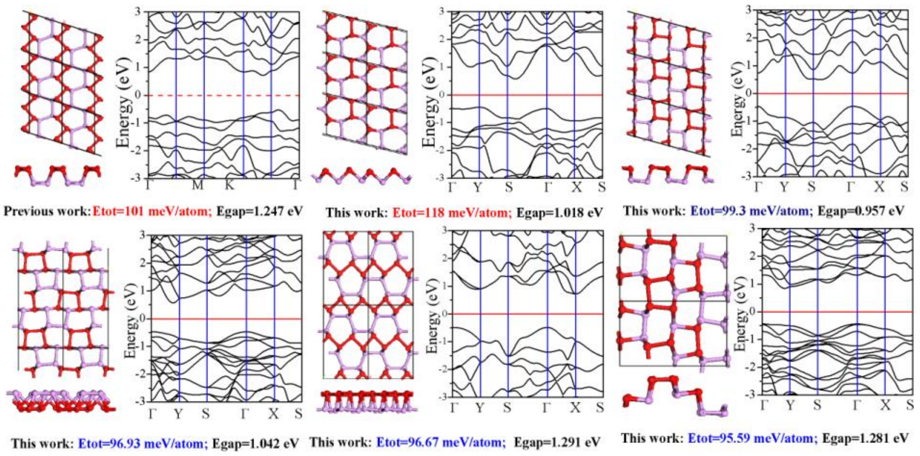

Fig. S6 The optimized crystal structures, relative energies (respect to **α-P**, meV/atom), band structures and band gaps of single-layered 5-7 phosphorene allotropes based on DFT-calculation.

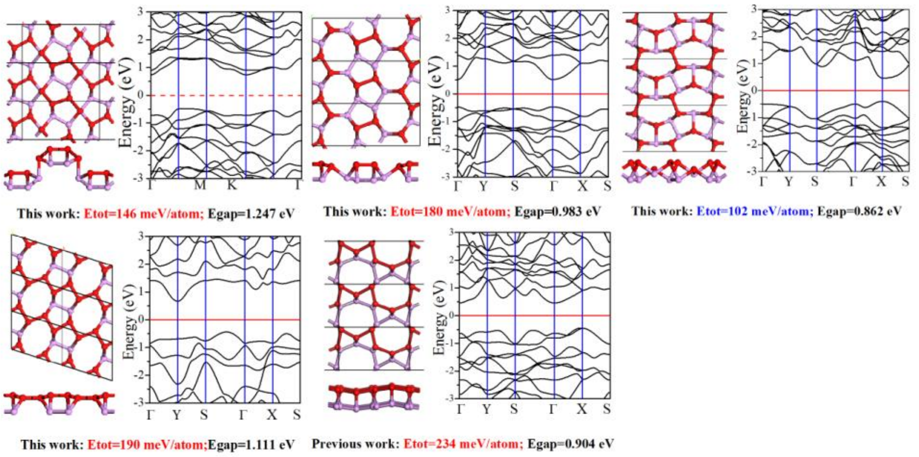

Fig. S7 The optimized crystal structures, relative energies (respect to **α-P**, meV/atom), band structures and band gaps of single-layered 5-8 phosphorene allotropes based on DFT-calculation.

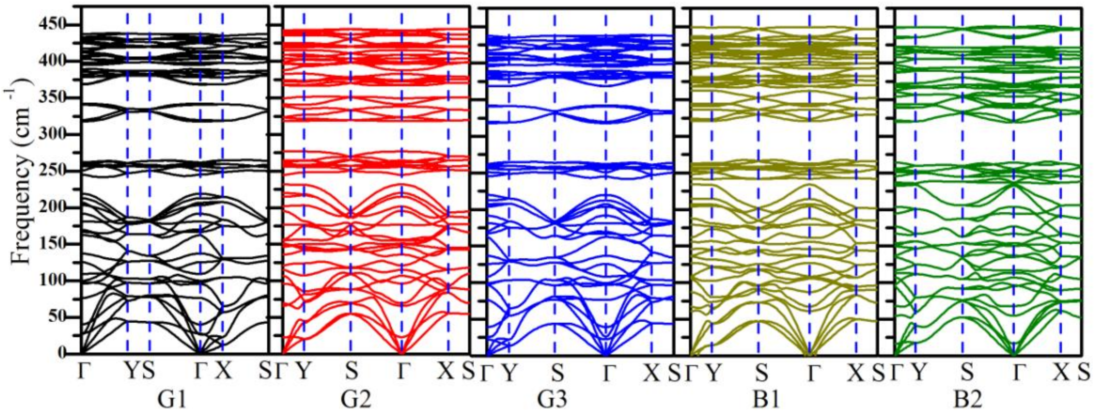

Fig. S8. Simulated phonon band structures of the five new phosphorene allotropes **G1, G2, G3, B1** and **B2** based on DFT-calculation.

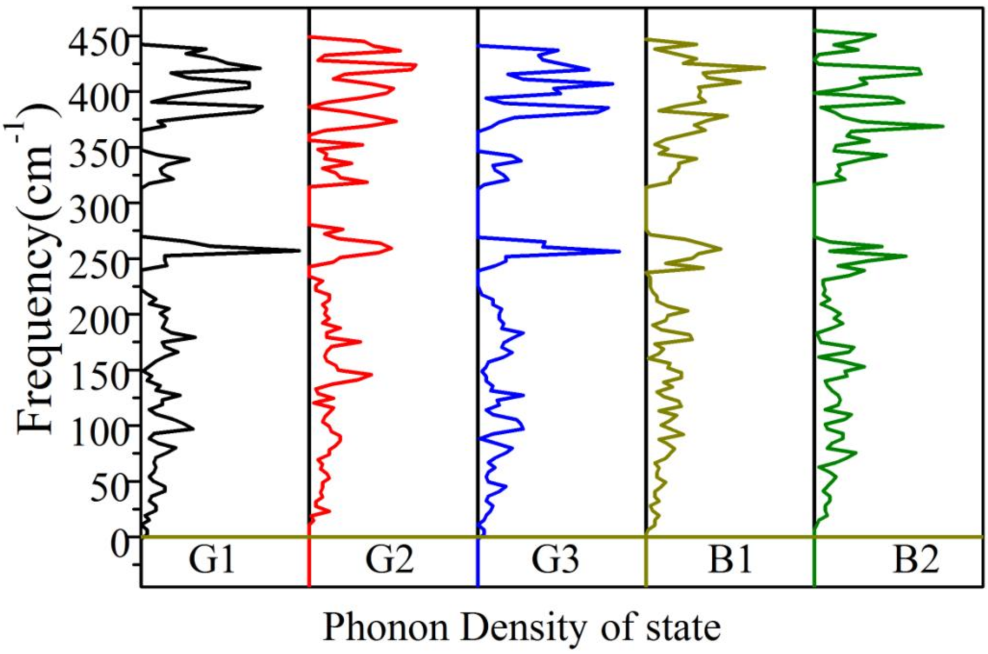

Fig.S9. Phonon density of states of allotropes **G1, G2, G3, B1** and **B2.**

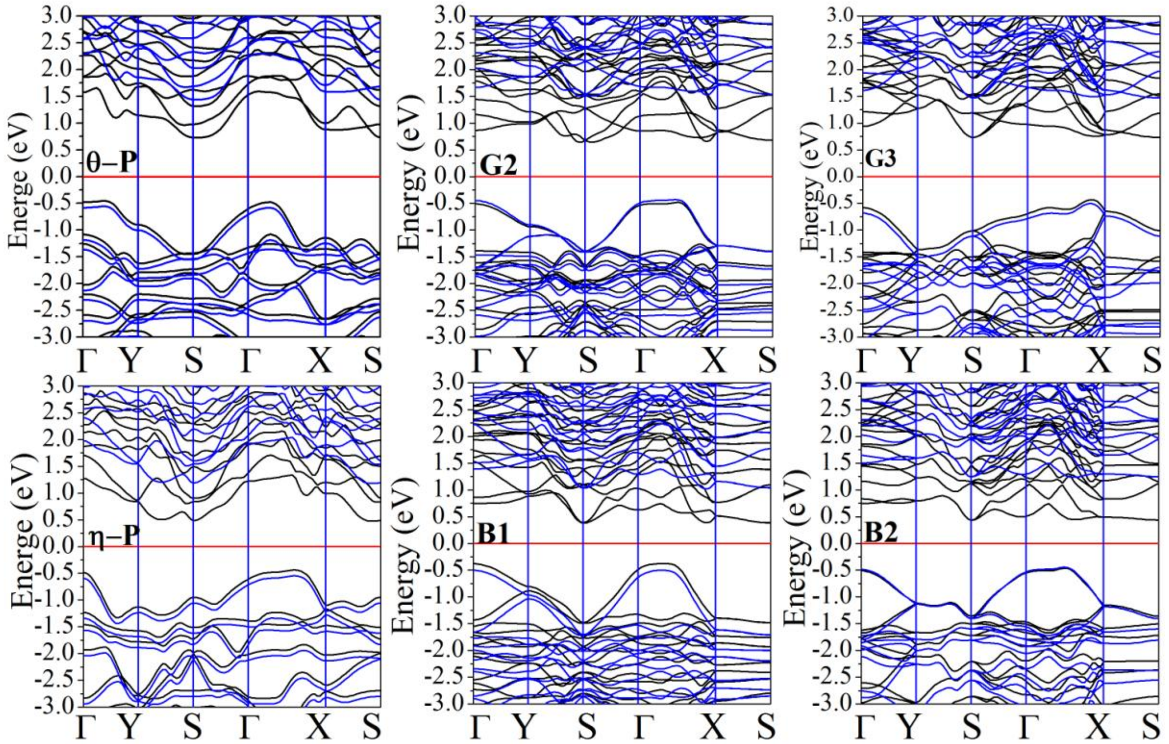

Fig. S10. Electronic band structures of allotropes **η-P**, **θ-P**, **G2, G3, B1** and **B2**, black solid lines are calculated form DFT method, blue solid lines are calculated form HSE06 method.

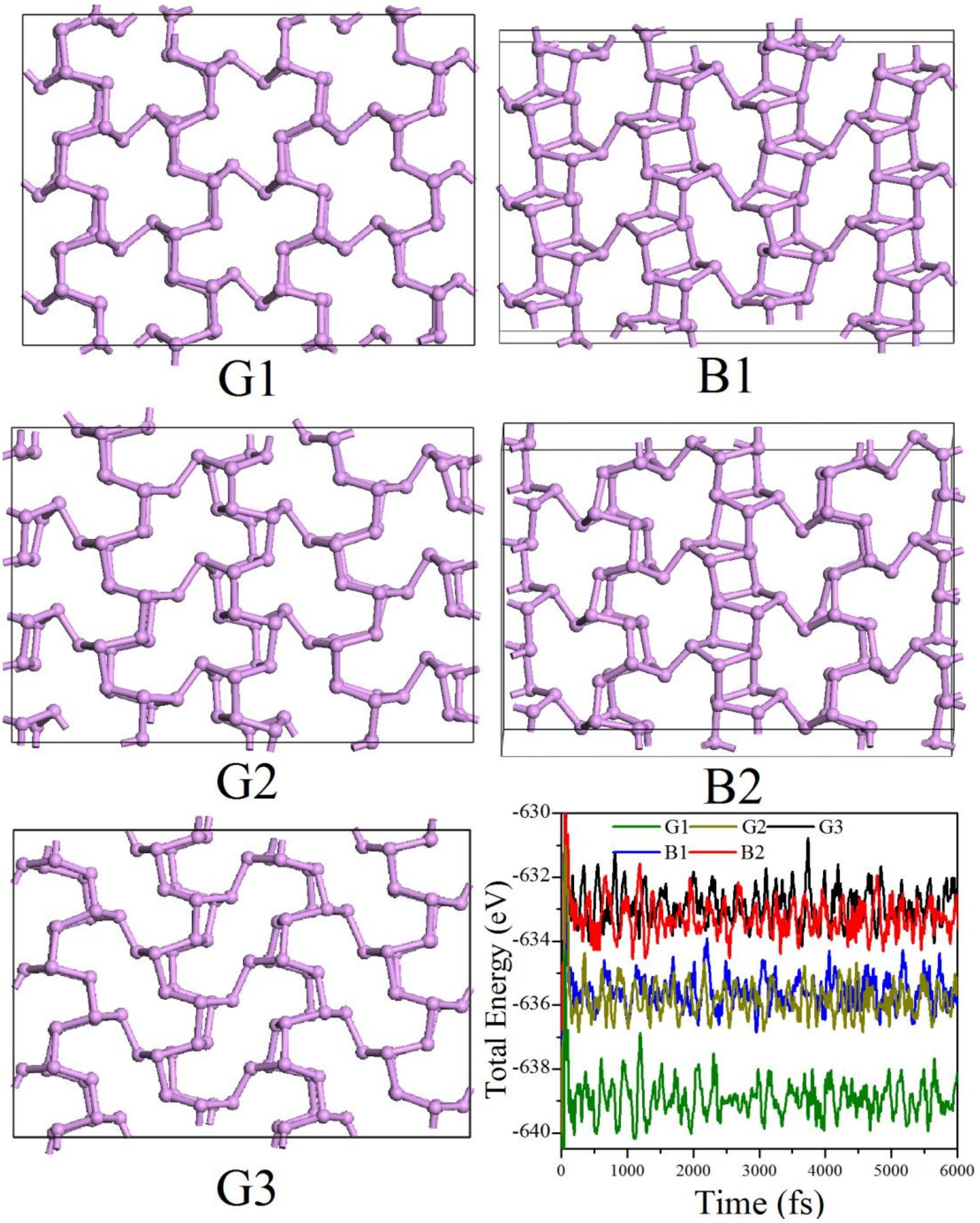

Fig. S11. Snapshot of the equilibrium structures at 300 K for bi-layered G1, G2, G3, B1 and B2 at the end of 6 ps of BOMD simulation. The BOMD simulation is performed in the cannonical ensemble with the temperature being controled at 300 K using the Nosè algorithm, with the time step of 2fs. The fluctuations of potential energy of the five new allotropes in the process of BOMD simulation.

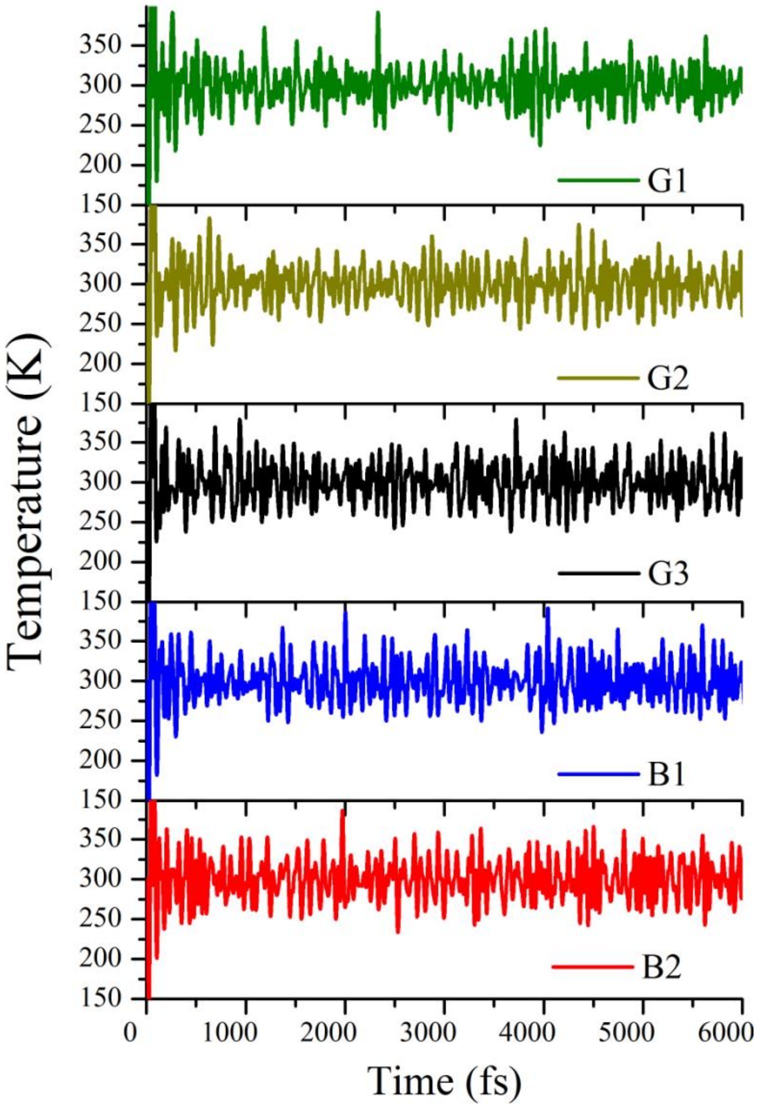

Fig. S12. The fluctuations of temperature of the five new allotropes in the process of BOMD simulation.

Table S1. Crystalline information of the bi-layered phosphorene allotropes from this work.

System name: G1 (this work) **Space group:** Pbam No.55

Lattice: a=11.849;b=5.792;c=25.000;α=90.00; β=90.00; γ=90.00

P1:	0.167	0.506	0.569
P2:	0.337	0.379	0.545
P3:	0.071	0.369	0.500

System name: G2 (this work) **Space group:** Pmc21 No.26

Lattice: a=25.000; b=12.144;c=5.532;α=90.00; β=90.00; γ=90.00

P1:	0.455	0.077	0.251
P2:	0.431	0.092	0.859
P3:	0.500	0.196	0.733
P4:	0.500	0.295	0.078
P5:	0.431	0.407	0.105
P6:	0.454	0.423	0.494

System name: G3 (this work) **Space group:** Pbam No.55

Lattice: a= 12.281; b=5.533;c=25.00; α= 90.00; β=90.00; γ=90.00

P1:	0.155	0.686	0.569
P2:	0.177	0.075	0.546
P3:	0.039	0.681	0.500

System name: B1 (this work) **Space group: Pc No.7**

Lattice: a=25.000;b=12.175; c=5.539; α=90.00; β=85.604; γ=90.00

P1:	0.430	0.081	0.068
P2:	0.520	0.076	0.033
P3:	0.539	0.095	0.430
P4:	0.404	0.085	0.687
P5:	0.466	0.199	0.509
P6:	0.476	0.306	0.837
P7:	0.405	0.407	0.746
P8:	0.427	0.423	0.344
P9:	0.518	0.422	0.306
P10:	0.544	0.412	0.683

System name: B2 (this work) **Space group:** Pc No.7

Lattice: a= 25.00; b=12.287; c=5.442; α=90.00; β=88.143; γ=90.00

P1:	0.490	0.047	0.329
P2:	0.430	0.165	0.495
P3:	0.565	0.149	0.274
P4:	0.457	0.329	0.382
P5:	0.547	0.321	0.364

System name: η-P (previous) **Space group:** P-1 No.2 **(**Nano. Lett., 17, 3557 (2015))

Lattice: a=25.00; b=6.351; c=5.149; α=77.07; β=89.78; γ=90.86

P1:	0.571	0.685	0.099
P2:	0.509	0.905	0.198
P3:	0.436	0.688	0.296
P4:	0.457	0.352	0.289

System name: θ-P (previous) **Space group:** Pm No.6 **(**Nano. Lett., 17, 3557 (2015))

Lattice: a=5.537; b=25.00; c=6.236; α=90.00; β=76.59; γ=90.00

P1:	0.250	0.500	0.386
P2:	0.332	0.569	0.161
P3:	0.731	0.546	0.132
P4:	0.313	0.545	0.822
P5:	0.886	0.500	0.585
P6:	0.929	0.569	0.793

Table S2. Crystalline information for the single-layered 6-6 phosphorene allotropes

System name: blue-P, β-P, chair **Space group:** P-3m1 No.164
Lattice: a=3.269;b=3.269;c=25.000;α=90.00; β=90.00; γ=120.00
P1: 0.667 0.333 0.524
References: PRL 112, 176802 (2014)

System name: black-P, α-P, stirrup **Space group:** Pmna No.53
Lattice: a= 3.306;b=25.000;c=4.509;α=90.00; β=90.00; γ=90.00
P1: 0.500 0.543 0.916
References: ACS nano., 8, 4033 (2014), Nat. Nano. 5, 372 (2014).

System name: red-P, tricycle **Space group:** Pbcm No.57
Lattice: a=25.000;b=8.989;c=3.294;α=90.00; β=90.00; γ=90.00
P1: 0.576 0.166 0.250
P2: 0.491 0.083 0.250
References: J. Phys.:Condens.Matter. 27, 265301 (2015)

System name: γ-P, boat-1 **Space group:** Pmmn No.59
Lattice: a=3.254;b=5.406;c=25.00;α=90.00; β=90.00; γ=90.00
P1: 0.500 0.215 0.530
References: PRL 113, 046804 (2014)

System name: δ-P, boat-2 **Sace group:** Pbcm No.57
Lattice: a=25.000;b=5.450;c=5.389;α=90.00; β=90.00; γ=90.00
P1: 0.457 0.548 0.461
References: PRL 113, 046804 (2014)

System name: θ_0-P, twist-boat **Space group:** Pcca No.54
Lattice: a=5.779;b=25.000;c=6.129;α=90.00; β=90.00; γ=90.00
P1: 0.602 0.531 0.908
References: ACS nano., 8, 12763 (2014)

Table S3. Crystalline information for the single-layered 4-7 phosphorene allotropes

System name: New **Space group:** P4bm No.100
Lattice: a=7.011;b=7.011;c=25.000;α=90.00; β=90.00; γ=90.00
P1: 0.115 0.385 0.569
P2: 0.261 0.500 0.495
References: this work

System name: New **Space group:** P-421m No.113
Lattice: a=7.585;b=7.585;c=25.00;α=90.00; β=90.00; γ=90.00
P1: 0.029 0.189 0.481
P2: 0.106 0.394 0.541
References: this work

System name: New **Space group:** P21/c No.14
Lattice: a=25.000;b=7.594;c=7.484;α=90.00; β=90.00; γ=90.00
P1: 0.524 0.099 0.921
P2: 0.465 0.322 0.947
P3: 0.525 0.499 0.811
References: this work

Table S4. Crystalline information for the single-layered 3-12 phosphorene allotropes

System name: New **Space group:** Pmmn No.59
Lattice: a=10.096;b=5.481;c=25.000;α=90.00; β=90.00; γ=90.00
P1: 0.896 0.500 0.441
P2: 0.844 0.296 0.514
References: this work

System name: New **Space group:** Cmm2 No.35
Lattice: a=10.702;b=5.371;c=25.00;α=90.00; β=90.00; γ=90.00
P1: 0.147 0.289 0.339
P2: 0.109 0.500 0.265
References: this work

System name: Kagome; 3-12 (N=2) **Space group:** P-3M1 No.164
Lattice: a=5.468;b=5.468;c=25.000;α=90.00; β=90.00; γ=120.00
P1: 0.529 0.471 0.543
References: ACS nano., 8, 12763 (2014);J. Phys.: Condens. Matter. 27, 255006 (2015);

Table S5. Crystalline information for the single-layered 4-8 phosphorene allotropes

System name: New **Space group:** P4/nmm No.129
Lattice: a=5.475;b=5.475;c=25.000;α=90.00; β=90.00; γ=90.00
P1: 0.211 0.289 0.543
References: this work

System name: 4-8 (N=0) **Space group:** P4/nbm No.125
Lattice: a=6.457;b=6.457;c=25.000;α=90.00; β=90.00; γ=90.00
P1: 0.147 0.353 0.475
References: ACS nano., 8, 12763 (2014)

System name: New **Space group:** P-4m2 No.115
Lattice: a=4.889;b=4.889;c=25.000;α=90.00; β=90.00; γ=120.00
P1: 0.500 0.245 0.528
References: This work

System name: ε-P **Space group:** P4212 No.90
Lattice: a=5.371;b=5.371;c=25.000;α=90.00; β=90.00; γ=90.00
P1: 0.257 0.341 0.543
References: Nano. Lett., 17, 3557 (2015)

System name: ζ-P **Space group:** P-1 No.2
Lattice: a=6.452;b=5.325;c=25.000;α=90.00; β=90.00; γ=90.00
P1: 0.084 0.264 0.488
P2: 0.341 0.369 0.544
P3: 0.584 0.232 0.487
P4: 0.840 0.143 0.544
References: Nano. Lett., 17, 3557 (2015)

Table S6. Crystalline information for the single-layered 5-7 phosphorene allotropes

System name: 5-7 (N=2) **Space group:** P2 No.3
Lattice: a=5.423;b=25.00;c=6.258;α=90.00; β=75.00; γ=90.00

P1:	0.137	0.519	0.597
P2:	0.296	0.437	0.619
P3:	0.298	0.445	0.991

References: ACS nano., 8, 12763 (2014)

System name: New **Space group:** P-1 No.2
Lattice: a= 6.297;b=25.00;c=6.205;α=90.00; β=76.44; γ=90.00

P1:	0.076	0.525	0.614
P2:	0.368	0.471	0.591
P3:	0.381	0.468	0.966
P4:	0.899	0.478	0.904

References: this work

System name: New **Space group:** P-1 No.2
Lattice: a=5.466;b=25.00;c=6.232;α= 90.00; β=74.94; γ=90.00

P1:	0.000	0.527	0.361
P2:	0.423	0.459	0.582
P3:	0.446	0.459	0.947
P4:	0.974	0.535	0.898

References: This work

System name: New **Space group:** P21/C No.14
Lattice: a=25.00;b=6.186;c=10.51;α=90.00; β=90.27; γ=90.00

P1:	0.472	0.137	0.004
P2:	0.534	0.389	0.016
P3:	0.541	0.452	0.226
P4:	0.541	0.826	0.210

References: This work

System name: New **Sace group:** Pba2 No.32
Lattice: a=6.228;b=10.138;c=25.00;α=90.00; β=90.00; γ=90.00

P1:	0.135	0.932	0.610
P2:	0.194	0.855	0.527
P3:	0.567	0.852	0.535
P4:	0.616	0.917	0.621

References: This work

System name: New **Space group:** P21 No.4
Lattice: a=6.192;b=10.242;c=25.00;α=90.00; β=90.06; γ=90.00

P1:	0.296	0.758	0.433
P2:	0.427	0.724	0.517
P3:	0.799	0.739	0.517
P4:	0.917	0.778	0.432

References: This work

Table S7. Crystalline information for the single-layered 5-8 phosphorene allotropes

System name: New **Space group:** P2221 No.17
Lattice: a= 25.00;b=4.679;c=9.487;α=90.00; β=90.00; γ=90.00
P1: 0.410 0.074 0.112
P2: 0.477 0.388 0.102
P3: 0.453 0.742 0.248
References: This work

System name: New **Space group:** P2221 No.17
Lattice: a=25.00;b=4.762;c=11.618;α=90.00; β=90.00; γ=90.00
P1: 0.439 0.100 0.085
P2: 0.507 0.409 0.088
P3: 0.462 0.727 0.194
References: This work

System name: New **Space group:** Pma2 No.28
Lattice: a=9.206;b=5.841;c=25.00;α=90.00; β=90.00; γ=90.00
P1: 0.046 0.179 0.514
P2: 0.250 0.176 0.463
P3: 0.250 0.549 0.486
P4: 0.056 0.672 0.439
References: This work

System name: New **Space group:** P2 No.3
Lattice: a=3.254;b=5.406;c=25.00;α=90.00; β=72.68; γ=90.00
P1: 0.156 0.533 0.825
P2: 0.169 0.459 0.520
P3: 0.336 0.521 0.149
References: This work

System name: 5-8 (N=2) **Sace group:** P21/m No.11
Lattice: a=5.479;b=9.097;c=25.00;α=90.00; β=91.45; γ=90.00
P1: 0.463 0.984 0.543
P2: 0.616 0.750 0.556
P3: 0.750 0.750 0.472
P4: 0.940 0.965 0.459
References: ACS nano., 8, 12763 (2014)
